# G-CSF plus azacitidine versus azacitidine alone for patients with high-risk myelodysplastic syndrome: academic, open label, randomized trial

**DOI:** 10.1038/s41408-022-00698-2

**Published:** 2022-07-07

**Authors:** Tomáš Stopka, Lubomír Minařík, Nina Dusilková, Michal Pešta, Vojtěch Kulvait, Martin Špaček, Zuzana Zemanová, Marta Kalousová, Anna Jonášová

**Affiliations:** 1 Haematology Clinic, General Faculty Hospital, Prague, Czech Republic; 2 BIOCEV, 1st Medical Faculty, Charles University, Vestec, Czech Republic; 3 Pathophysiology, 1st Medical Faculty, Charles University, Prague, Czech Republic; 4 Faculty of Mathematics & Physics, Charles University, Prague, Czech Republic; 5 Cytogenetics, 1st Medical Faculty, General Hospital and Charles University, Prague, Czech Republic; 6 Institute of Medical Biochemistry and Laboratory Diagnostics, General Faculty Hospital, Prague, Czech Republic

**Keywords:** Myelodysplastic syndrome, Phase III trials

## Abstract

GA trial is registered under EudraCT#: 2013-001639-38.

**Dear Editor,**

Myelodysplastic syndromes (MDS) emerge as a disorder of stem cell differentiation and maturation, resulting in peripheral cytopenias and eventual progression to acute myeloid leukemia (AML). Numerous clonal genetic abnormalities together with varying degrees of cytopenias and myeloblast (MB) accumulation are the basis for the revised International Prognostic Scoring System (IPSS-R) [1]. Gradual disease progression worsens survival and is an indication for starting treatment with hypomethylating agents (HMAs) such as 5-azacytidine (AZA) or decitabine, in some cases as a bridge to transplantation or as continuous therapy until failure for patients who are not transplant candidates. Compared to conventional chemotherapy, AZA treatment prolongs survival in both higher-risk MDS and oligoblastic (20–30%) MDS/AML (24.5 vs 16 months) [2–4]. AZA induces more sustained hematologic responses, but does not lead to durable remissions and most patients eventually progress and fail therapy. To improve efficacy, new agents such as Venetoclax [5, 6], Pevonedistat [7] or Panobinostat [8] have been tested in combination with the standard AZA regimen, while others (Sabatolimab, Magrolimab, IDH1/IDH2 inhibitors) are being tested. G-CSF (granulocyte colony stimulating factor) activates myeloid gene transcription in stem cells if added prior to HMA [9, 10]. G-CSF is used in MDS for neutropenic complications. In older pre-treated patients with breast cancer or non-Hodgkin’s lymphoma, filgrastim administration is a risk for MDS development [11, 12]. G-CSF may act on MDS cells by activating their cell cycle and differentiation, leading to selection against G-CSF receptor signaling [13]. Our clinical retrospective data of 162 HR-MDS patients treated with AZA associated higher G-CSF consumption (*N* = 35) with a lower incidence of grade (Gr) 4 neutropenia, and consequently longer overall survival (OS, median 27.4 vs 18 Mo, *p* = 0.017, Supplementary Material SM1). We investigated the effect of G-CSF on AZA efficacy by academic prospective randomized trial (SM2).

A total of 76 patients with high risk MDS and MDS/AML bellow 30% myeloblasts ineligible for transplantation were enrolled in the GA study from February 6, 2017 to December 31, 2021. Patients were randomized into arm A (AZA monotherapy) and arm GA (G-CSF + AZA). Study objectives included response rate, OS, progression-free survival (PFS), duration of response and safety of administration (detailed in SM2-3). Three patients died early and were neither randomized nor started therapy and 3 randomized patients into arm A died during the first AZA cycle, thus 70 patients in GA (*N* = 39) and A (*N* = 31) arms were analyzed. The median age and male to female ratio in the GA arm were 73 years and 23:16 (59% males) versus 74 years and 15:16 (48%) in arm A. The data of the cohort tested including subtypes and IPSS-R are presented in SM4-5. Statistics is described in SM6-7. Patients in both arms had comparable hematology findings (SM5), but they were not perfectly balanced; for example, arm A had more bone marrow blasts and arm GA had a higher proportion of patients with t-MDS. Patients with EB2 and MDS/AML had a slightly higher mutational burden compared to other high-risk patients with MB counts below 10% (SM8-9).

AZA administration was standard 7-day (75 mg/m2, SM2), G-CSF was administered 2 days prior the 1st dose of AZA and 2 days prior the 6th dose of AZA (SM2) at dose 5 μg/kg of body weight. The median number of AZA cycles was 8 (range 1–40). The efficacy of G-CSF administration was verified using the CD64 biomarker [14] on granulocytes (SM10) and by measuring plasma G-CSF levels (SM11) after the first cycle of therapy. OS and therapeutic response between the arms were assessed at multiple time points using longitudinal multivariate data analysis and a Joint model including time-constant (sex, input DNA variants, NGS analysis described in SM12) and time-varying (laboratory data) parameters. The Cox proportional hazards model containing time-varying covariates together with the ordinal multilevel logistic mixed model provide a plausible statistical framework for the aforementioned evaluation (Table 1). Although the Kaplan–Meier plot is crossed between the arms at the end of follow-up in terms of OS (Fig. 1), this view involves only univariate empirical analysis. For the designed arms, the median OS times are 443 days (14.8 months) in the GA arm and 402 days (13.4 months) in the A arm (95% CI: [362,737] and [147,580] days, respectively) (*p* = 0.300, Cochran-Mantel-Haenszel logrank test). However, there are confounding effects of G-CSF injections in particular, which stem from the fact that patients in both arms could receive G-CSF for ethical reasons in the event of febrile neutropenia (SM13). Thus, there are also patients in arm A who received G-CSF (*N* = 6, 19%). In addition, there is a trend towards more frequent use of G-CSF upon HMA failure in Year 2, and the difference between arms in terms of the number of G-CSF injections equalizes from a 4:1 to a 2:1 ratio. Thus, patients in the GA arm have a lower risk of death and GA treatment significantly prolongs OS (*p* = 0.0297). In contrast, for arm A, the risk of death is higher up to approximately 13 cycles of therapy, where the quadratic parabola of the relationship with G-CSF applications reaches its extreme. Such a declining-rising effect of the number of G-CSF cycles on survival is depicted in SM14. After roughly one year of HMA, when there is a gradual failure of therapy and an increase in infectious complications, G-CSF is a rather neutral parameter for survival. In addition to G-CSF, detected DNA variants also influence OS: negative predictors are *DNMT3A* mutations (*p* = 0.0131), *ETV6* (*p* = 0.0012), *EZH2* (*p* = 0.0044), positive: *SF3B1* (*p* = 0.0005). Male patients tend to have a longer OS (*p* = 0.0041) while Gr4 neutropenia indicates a shorter OS (*p* = 0.0229). Predicted survival curves include SM15-16.

**Table 1:** Fitted joint model for the overall survival on the GA vs A and the response to the G-CSF therapy of the GA vs A arm.

	Coefficient	SE	95% CI for coefficient	Hazard ratio^a^/Odds ratio^b^	95% CI for HR/OR	*P* value
Cox PH model for OS time^a^				Hazard ratio		Score (logrank overall) test < 0.0001
Arm (GA vs A)	−0.4516	0.2078	−0.8589, −0.0444	0.6366	0.4236, 0.9566	**0.0297**
Number of G-CSF cycles (1 cycle increase)	0.0885	0.0395	0.0110, 0.1660	1.0926	1.0111, 1.1806	**0.0252**
(Number of G-CSF cycles)^2	−0.0033	0.0013	−0.0057, −0.0008	0.9967	0.9943, 0.9992	**0.0100**
Gender (Male vs Female)	−0.5944	0.2073	−1.0007, −0.1880	0.5519	0.3676, 0.8286	**0.0041**
Neutropenia Gr4 in 4 cycles (Yes vs No)	0.5639	0.2479	0.0780, 1.0498	1.7575	1.0811, 2.8570	**0.0229**
* DNMT3A* (Mutated vs Unmutated)	0.6185	0.2492	0.1299, 1.1069	1.8561	1.1388, 3.0251	**0.0131**
* ETV6* (Mutated vs Unmutated)	1.2855	0.3961	0.5091, 2.0618	3.6164	1.6638, 7.8605	**0.0012**
* EZH2* (Mutated vs Unmutated)	1.4236	0.5004	0.4429, 2.4043	4.1519	1.5572, 11.0701	**0.0044**
* SF3B1* (Mutated vs Unmutated)	−1.8870	0.5398	−2.9450, −0.8290	0.1515	0.0526, 0.4365	**0.0005**
Ordinal multivariate logistic mixed model for response to the therapy^b^				Odds ratio		Likelihood ratio (overall) test < 0.0001
Arm (GA vs A)	−1.3744	0.4139	−2.1858, −0.5631	0.2530	0.1124, 0.5694	**0.0009**
G-CSF injections / 4-cycle (1 inj. increase)	0.3443	0.0659	0.2152, 0.4734	1.4110	1.2401, 1.6050	**<0.0001**
(Number of G-CSF inj. per 4-cycle)^2	−0.0085	0.0022	−0.0128, −0.0043	0.9915	0.9873, 0.9957	**<0.0001**
MB% PB	−0.1501	0.0445	−0.2373, −0.0629	0.8606	0.7888, 0.9391	**0.0007**
PLT	0.0499	0.0087	0.0329, 0.0670	1.0513	1.0335, 1.0690	**<0.0001**
HB	0.0073	0.0018	0.0038, 0.0108	1.0073	1.0038, 1.0110	**<0.0001**

*P* values in bold (far right)

*SE* indicates standard error, *CI* confidence interval.

^a^A positive (negative) coefficient estimate in the time-varying Cox PH model indicates a higher (lower) risk of death and therefore a shorter (longer) OS.

^b^A positive coefficient estimate in the ordinal multivariate logistic mixed model indicates a remission response to treatment rather than progression.

**Fig. 1:**
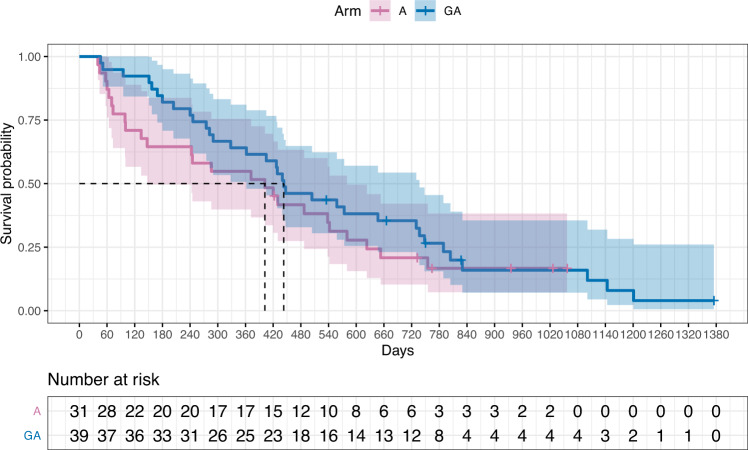
Kaplan–Meier plot. Survival probability versus time (in days) for both treatment arms (GA vs A) of the clinical trial.

Response to treatment was assessed according to IWG criteria [15] (SM17, Table1). Overall response rate (ORR, GA vs A) was 77% vs 61% (*p* = 0.000899), CR 31% vs 23% (*p* = 0.575), PR 23% vs 23% (*p* = 0.554), SD with HI 18% vs 0% (*p* = 0.473), SD without HI 8% vs 13% (*p* = 0.739). Progression-free survival (PFS, GA vs A) was 9.7 vs 6.1 months (95% CI: [254,831] and [64,208] days, respectively) (*p* = 0.09, Cochran-Mantel-Haenszel logrank test). When in the first four cycles of AZA, many patients belonging to the GA arm responded with CR/PR/HI (*N* = 28, 72%), whereas twice as few in the A arm did (*N* = 14, 45%). This is particularly important for those patients, who are at increased risk of infectious and other complications associated with cytopenias during the initial cycles of AZA; on the other hand, achieving a better response gives the chance of a longer OS. Hemoglobin (HB) and platelets (PLT) have a positive expected effect on treatment response (*p* < 0.0001 for each), while peripheral blood MBs have a negative expected effect (*p* = 0.0007, SM18).

One very important parameter in this study was the rate of progression to AML during therapy in relation to G-CSF administration (SM19). Progression to AML was comparable as observed in 20 patients in the GA arm (52%) vs. 21 patients in the A arm (68%) (*p* = 0.968). Time to progression to AML was also comparable: 9.8 months in the GA arm vs. 8.9 months in the A arm (*p* = 0.450). This was comparably observed in both arms at each restaging (SM17). Thus, we found no effect of the addition of G-CSF on progression to AML throughout the HMA treatment period. There was also no difference between the arms in terms of treatment toxicity assessment (SM20). Regarding infectious complications, infection-related mortality in the GA arm during the first 4 cycles of therapy was lower compared to A arm (8 vs 29%, see SM21).

Clinical testing of G-CSF therapy, inspired by preclinical effects prior to the use of HMA [9, 10] has shown that G-CSF prior AZA is useful in the very early stages of therapy by inducing more durable responses and thus avoiding complications associated with cytopenia, and secondly by allowing the administration of AZA in the introduction at full dose and without prolonging the intervals between treatments, which is often caused by infectious complications. Both arms have experienced therapeutic failure of AZA at later time points comparably and thus the use of G-CSF is unable to prevent therapeutic failure of AZA. Interestingly, responses in the GA arm occurred relatively early in the first four cycles of AZA (31 in GA vs 18 in A; Fisher exact probability test gives *p* = 0.0260), which was not observed in the A arm, where a significant proportion of patients died due to infectious complications. Furthermore, the time to response is significantly lower in the GA arm compared to the A arm (*p* = 0.00184). Moreover, the positive effect of G-CSF is reinforced by the fact that the presence of Gr4 neutropenia is associated with significantly shorter OS (SM15-16).

Our primary objective of increasing treatment response and survival in the GA versus A arm was confirmed, particularly in patients with initial neutropenia in the first year of HMA treatment. We did not detect an effect of G-CSF on progression to AML, which is also significant. Thus, the administration of G-CSF prior to AZA represents an improvement to the standard AZA regimen in patients with high-risk MDS and oligoblastic AML.

## Supplementary information

Supplementary Information

## Data Availability

Original data and protocols are available to other investigators upon request.
