# Supplementary material for: G-CSF plus azacitidine versus azacitidine alone for patients with high-risk myelodysplastic syndrome: academic, open label, randomized trial
Source: Blood Cancer J. 2022 Jul 7;12(7):105. doi: 10.1038/s41408-022-00698-2 (PMC9262924; doi:10.1038/s41408-022-00698-2)
Supplement: Supplementary file 1 — Supplementary Information [file 41408_2022_698_MOESM1_ESM.pdf]

SUPPLEMENTARY MATERIAL SM1-SM21

Supplementary Material SM1: Based on the Prague General Hospital registry analysis of the AZA therapy involving 162 HR-MDS patients we observed that the AZA-treated patients with use of G-CSF (N=35; median of 3 injections per cycle) had significantly longer OS (Log-rank test, median 27.4 vs 18 months, p=0.017, HR 0.5922, 95CI 0.3849-0.9112).

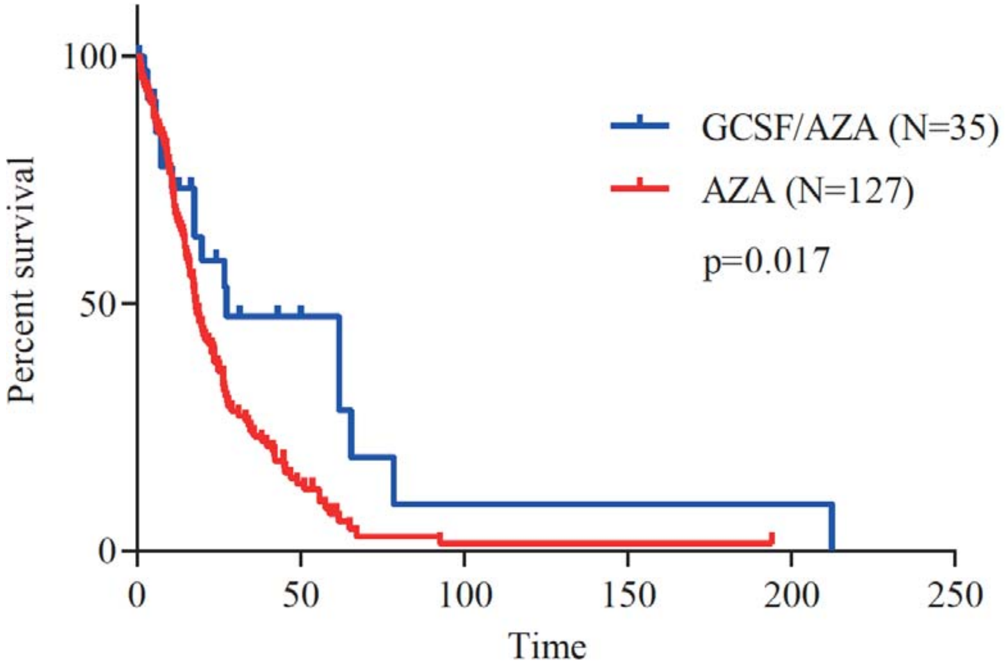

Supplementary Material SM2: Trial design and End points

The study titled "Contribution to the verification of the efficacy of adding granulocyte colony-stimulating factor (G-CSF) to 5-Azacitidine therapy in patients with high-risk myelodysplastic syndrome" was approved by the State Institute for Drug Control in the Czech Republic on December 6, 2016 under EudraCT#: 2013-001639-38 in accordance with ethical standards and the Helsinki Declaration of 1975 (as revised in 2008). The institutional ethics committee approved the study (#263/13S-IV) on April 21, 2016. The study included patients with higher risk MDS (Int-2 and high risk according to IPSS criteria (1)), MDS/AML with less than 30% MB and CMML II (according to 2016 WHO criteria), all > 65 years of age, not eligible for allo-HSCT or intensive chemotherapy and not previously treated with HMA. The dose and schedule of AZA administration were standard: 75 mg/m2 for seven days (5+2+2) subcutaneously within a 28-day cycle. G-CSF (Filgrastim, an unpegylated, recombinant cytokine, Neupogen from Amgen or Zarzio from Sandoz GmbH) was administered subcutaneously on day -2 (2 days before the 1st dose of AZA) and day +6 (the day following the 5th dose of AZA) at a dose of 30 MIU in patients with a body weight below 80 kg or 48MIU in patients with a body weight above 80 kg. The application schedule for the GA arm is shown in the figure below:

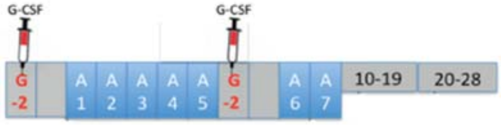

The main objectives were to prolong patient survival, to prolong the transformation to AML, to reduce the incidence of infections and hospitalisation time, and to achieve transfusion independence. Secondary objectives were to evaluate the association of clinical and genetic (somatic variants) variables within a specific study arm. Each patient signed an informed consent form and consented to DNA analysis by sequencing. Exclusion criteria were previous HMA treatment, eligibility for allo-HSCT, adherence to contraceptive measures, known hypersensitivity to study drugs, and failure to follow study guidelines.

The full study protocol is available on written request. The study is academic, which means that the academic institutions paid the salaries and wages of the investigators and the insurance of the patients. The molecular studies were sponsored in part by the grants listed in the Acknowledgments section. All authors had full access to all data in the study and accept responsibility for submitting it for publication.

Supplementary Material SM3: Efficacy and safety assessments

Complete blood count (CBC) and blood biochemistry were monitored in each cycle. Restaging analysis and response assessment included bone marrow collection for cytology, flow cytometry and cytogenetic evaluation every four cycles of AZA. BM analysis was also performed when progression was suspected, such as new cytopenias, new need for transfusion, or accumulation of MB in peripheral blood. Bone marrow aspirate analysis included flow cytometry, cytology and cytogenetic evaluation. Histological examination was performed at the time of diagnosis from the trephine biopsy. IPSS-R was determined before initiation of AZA treatment. Randomization was performed by lottery. Adherence to AZA therapy with G-CSF at d-2 and d+6 was designated as arm GA and adherence to standard AZA therapy was designated as arm A. Discontinuation of therapy was allowed at disease progression to AML or at patient request. Response was defined according to the IWG 2006 criteria (2), and in the case of this study, response was defined as either hematological improvement (HI), partial (PR), or complete remission (CR).

Clinical and laboratory monitoring of blood counts and baseline biochemistry (urea, creatinine, bilirubin, liver function tests and c-reactive protein) was performed weekly during the first 2 cycles of AZA and monthly after the 3rd cycle of AZA. For grade 4 cytopenias, AZA administration was delayed until recovery. Reduction of the AZA dose to 75% was allowed if the CBC had not recovered after 2 weeks of deferral. The occurrence of febrile neutropenia or any hospitalization was recorded as a serious adverse event (SAE).

Supplementary Material SM4:
Description of the study cohort. Parameters in the left column represnt numbers of patients (N), age, Male/Female ratio, MDS subtype, and tMDS for GA vs A arm.

**GA-MDS/2013 Cohort Description**	**GA**	**A**	**Overall**
Patients (N)	39	31	70
Age (Median, years)	73	74	73
M:F ratio	23:16	15:16	38:32
MLD (N)	2	3	5
EB-1 (N)	14	3	17
EB-2 (N)	15	14	29
AML (N)	7	9	16
CMML-1/2 (N)	1	2	1
Therapy related MDS (N)	10	4	14

Supplementary Material SM5: Description of the study parameters of CBC and BM on the left for all patients (left column) and in GA vs A arm. N states for a patient number, % percent.

GA-MDS/2013	GA	A	Overall
WBC (x10E9/l)	3·9	6·2	4·7
Hemoglobin (g/l)	91·6	94·9	93·2
Platelets (x10E9/l)	103·2	75·1	90·1
Abstolut neutrophil count	1·9	2·5	2·1
Lactate dehydrogenase (μkat/l)	4·9	4·3	4·4
Ferritin (μg/l)	1343·9	1017·6	1199·5
Peripheral blood myeloblasts (%)	4·5	6·2	5·2
Bone marrow myeloblasts (%)	11·4	16·1	13·5
Bone marrow fibrosis (Grade)	1·3	1·2	1·2
Transfusion dependent (N)	18	30	48
IPSS-R (Median)	6·5	5·5	6
IPSS-R Cytogenetic Score (Median)	3	1·5	2

Supplementary Material SM6: Statistical methods

One of the main objectives is to examine the impact of laboratory and clinical data during AZA treatment on overall survival and subsequently their impact on response in the GA vs A arm. We use a common model for OS in the GA vs A arm and response to a given therapy. Two types of covariates are included in the joint model: time-constant (e.g., sex or DNA variant represented by the 0-1 indicator) and time-varying (e.g., WBC or MB level for a specific time point). A stratified Cox proportional hazards (PH) model containing time-varying covariates, with strata consisting of dichotomous patient age (threshold 70 years), together with an ordinal multilevel logistic mixed model, provides a plausible statistical framework. Thus, all clinical data as well as the results of peripheral blood, biochemistry, flow cytometry, restaging and mutation analyses were evaluated with a joint statistical model of multivariate longitudinal and survival data developed previously (3). Such a stochastic framework is extended (4) such that OS and ordered multilevel treatment response are modeled together.

Supplementary Material SM7: Sample size calculation

The sample size calculation is based on power analysis to compare survival curves between the two arms (GA vs A) according to the Cox proportional hazards model for clinical (randomized) trials, which is an implementation of a previously described sample size calculation method (5). This method was originally proposed by Freedman (6). The power to detect the size of the risk ratio is assumed to be 0.90, the significance level (Type I error rate) is assumed to be 0.05, the proportion of patients in the GA arm versus the A arm is assumed to be 1.30, the probability of failure in the GA arm over the maximum study duration is assumed to be 0.30, the probability of failure in the A arm over the maximum study duration is assumed to be 0.45, and the postulated risk ratio is assumed to be 0.25. Then the expected number of patients enrolled in the GA arm is 40, while the expected number of patients in the A arm is 31.

Supplementary Material SM8: Overview of MDS associated variants in diagnostic BM samples prior therapy for each arm. The most frequently mutated gene is *TET2* on the top.

	GA	A	Overall
**TOTAL**	**85**	**55**	**140**
TET2	13	7	20
RUNX1	10	6	16
ASXL1	4	7	11
DNMT3A	5	3	8
STAG2	4	4	8
TP53	6	2	8
SRSF2	4	3	7
SF3B1	3	4	7
IDH2	2	4	6
CEBPA	2	4	6
IDH1	4	1	5
BCOR	5	0	5
EZH2	2	2	4
JAK2	1	3	4
KMT2A	4	0	4
BCORL1	3	0	3
CUX1	2	1	3
KDM6A	2	1	3
CSF3R	2	1	3
RAD21	0	2	2
NPM1	2	0	2
CBL	2	0	2
ETV6	2	0	2
CDKN2A	1	0	1

Supplementary Material SM9: Overview of MDS associated variants prior therapy in MLD, EB1 (<10% bone marrow myeloblasts) vs EB2, MDS/AML (>10% bone marrow myeloblasts) patients. Data indicate higher mutation load of driver genes in the group of patients with higher tumor burden.

	**<10% BM blasts**	**>10% BM blasts**	**Overall**
**TOTAL**	**41**	**99**	**140**
TET2	8	12	20
RUNX1	6	10	16
ASXL1	1	10	11
DNMT3A	2	6	8
STAG2	2	6	8
TP53	4	4	8
SRSF2	2	5	7
SF3B1	2	5	7
IDH2	0	6	6
CEBPA	1	5	6
IDH1	1	4	5
BCOR	1	4	5
EZH2	2	2	4
JAK2	1	3	4
KMT2A	3	1	4
BCORL1	0	3	3
CUX1	1	2	3
KDM6A	0	3	3
CSF3R	1	2	3
RAD21	0	2	2
NPM1	0	2	2
CBL	1	1	2
ETV6	2	0	2
CDKN2A	0	1	1

Supplementary Material SM10: CD64 expression determined by flow cytometry using Alexa 750 signal in patients belonging to GA or A arm. Measurement was performed prior the first G-CSF dose (before) and following the second G-CSF dose (after) during the first AZA cycle. t-test, $p<0.01$, 2 asterisks. Values of the same patient at two different time points are indicated by a line.

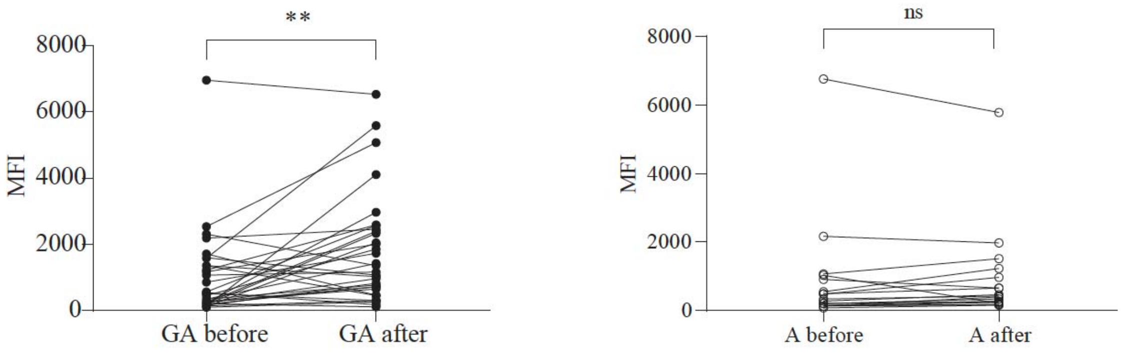

Supplementary Material SM11: Plasma G-CSF level as detected by flow cytometry. Two timepoints are compared: before (day -3) prior AZA start vs after (day 9) of the first cycle of GA (17.7 vs 50.17 pg/ml, p = 0.0003). Arm AZA was not significant ragarding G-CSF level (not shown).

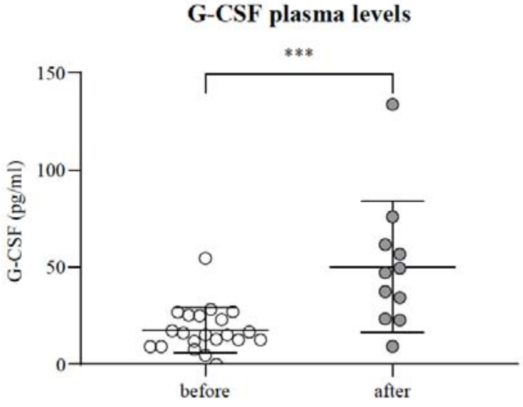

Supplementary Material SM12: Molecular analyses

Upon approval by the ethics committee and informed consent signed by each patient, it was feasible to sequence genomic DNA from BM mononuclear cells by next-generation sequencing (NGS) using the MDS NEB-Next 33 gene panel (New England Biolabs, Ipswich, MA, USA), which contains 33 frequently mutated MDS genes (7). NGS data were processed using an "in house" approach employing data quality assessment (FastQC, FastQ screen, multiQC). After removal of unwanted artificial sequences, mapping to the genome (GRCh38) was performed using alignment tools (BWA MEM). VarScan2 software was used to detect variants (8). To ensure the quality of variant detection, we only consider a region with a variant coverage of more than 50 reads per base with a variant frequency greater than 10% as variant detection. Subsequently, the identified variants were annotated using the dbSNP and ClinVar databases and their effect on the produced protein was evaluated using the SNPEff tool. A standard FISH-MDS panel was used to assess chromosomal aberrations.

Supplementary Material SM13: G-CSF and its administration in both arms.

In particular, for febrile neutropenia, 21 (54%) patients in the GA arm received an average of 4 doses of G-CSF over and above the 2 planned doses of G-CSF. Similarly, in arm A, 6 (20%) patients received a median of 2 G-CSF injections per AZA cycle in the case of febrile neutropenia. While the difference in total G-CSF consumption was indeed statistically different ($p<0.0001$, t test), G-CSF consumption for febrile neutropenia was similar between the two arms ($p=0.41$, t test). In the first year (or 12 cycles), the total number of G-CSF administrations was 968 vs 235 and in the second year (>12 cycles of therapy) 185 vs 71. The relative number (%) of febrile complications requiring antibiotic therapy in the first year was 54% vs 45% whereas in the second year febrile complications were 27% vs 25%.

Supplementary Material SM14: Predicted OS (y-axis) over time (x-axis, days) with respect to the treatment arm from a Cox PH model with time-varying covariates for a male patient (who received 4, 16, or 32 cycles of G-CSF) with Gr4 neutropenia and no mutated genes.

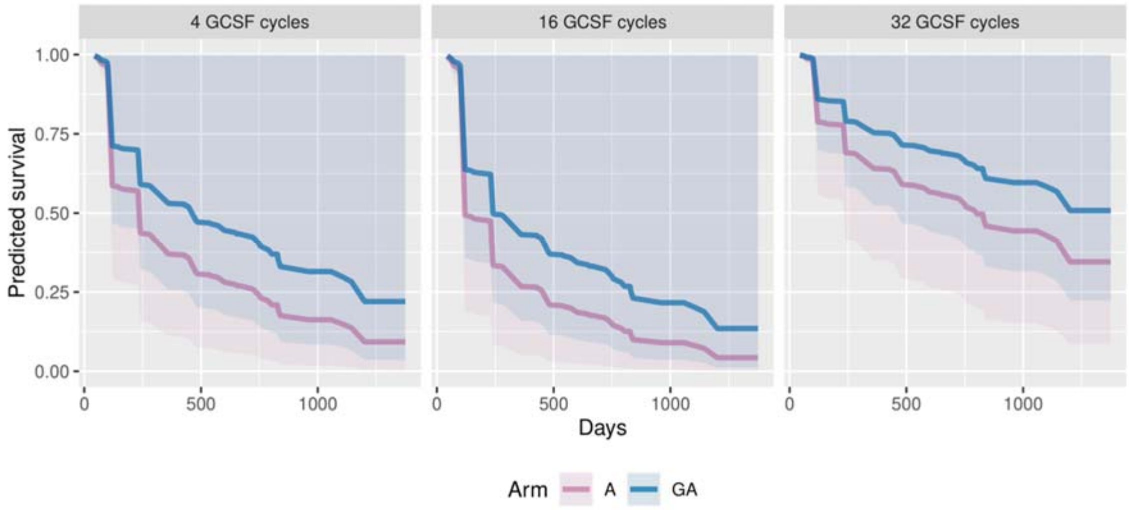

Supplementary Material SM15: Predicted OS (y-axis) over time (x-axis, days) with respect to the treatment arm from a Cox PH model with time-varying covariates for a male patient (who received 20 cycles of GCSF) with Gr4 neutropenia and no mutated genes.

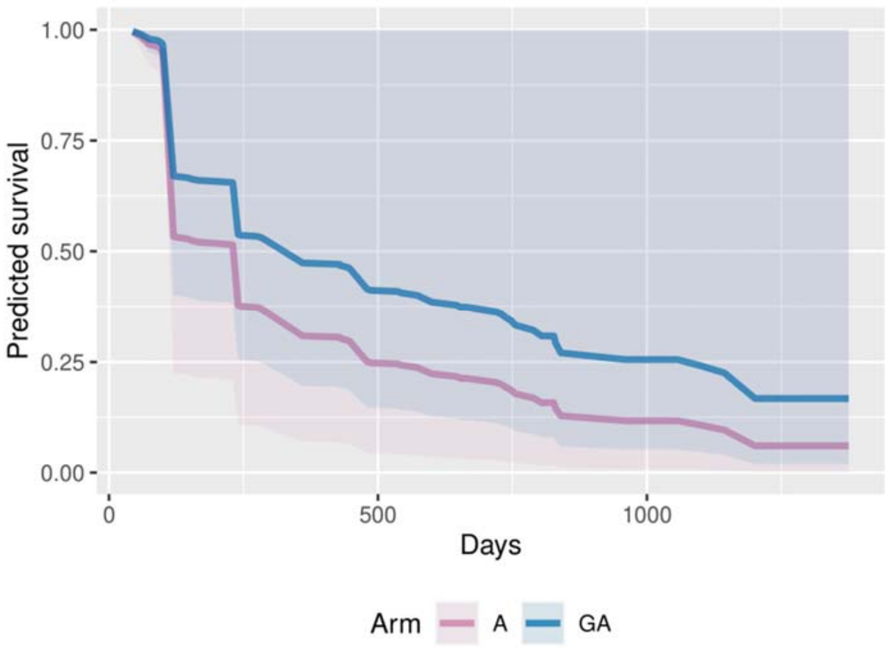

Supplementary Material SM16: Predicted OS (y-axis) over time (x-axis, days) with respect to the treatment arm from the Cox PH model with time-varying covariates under the conditions shown at the top and on the right side of each survival plot.

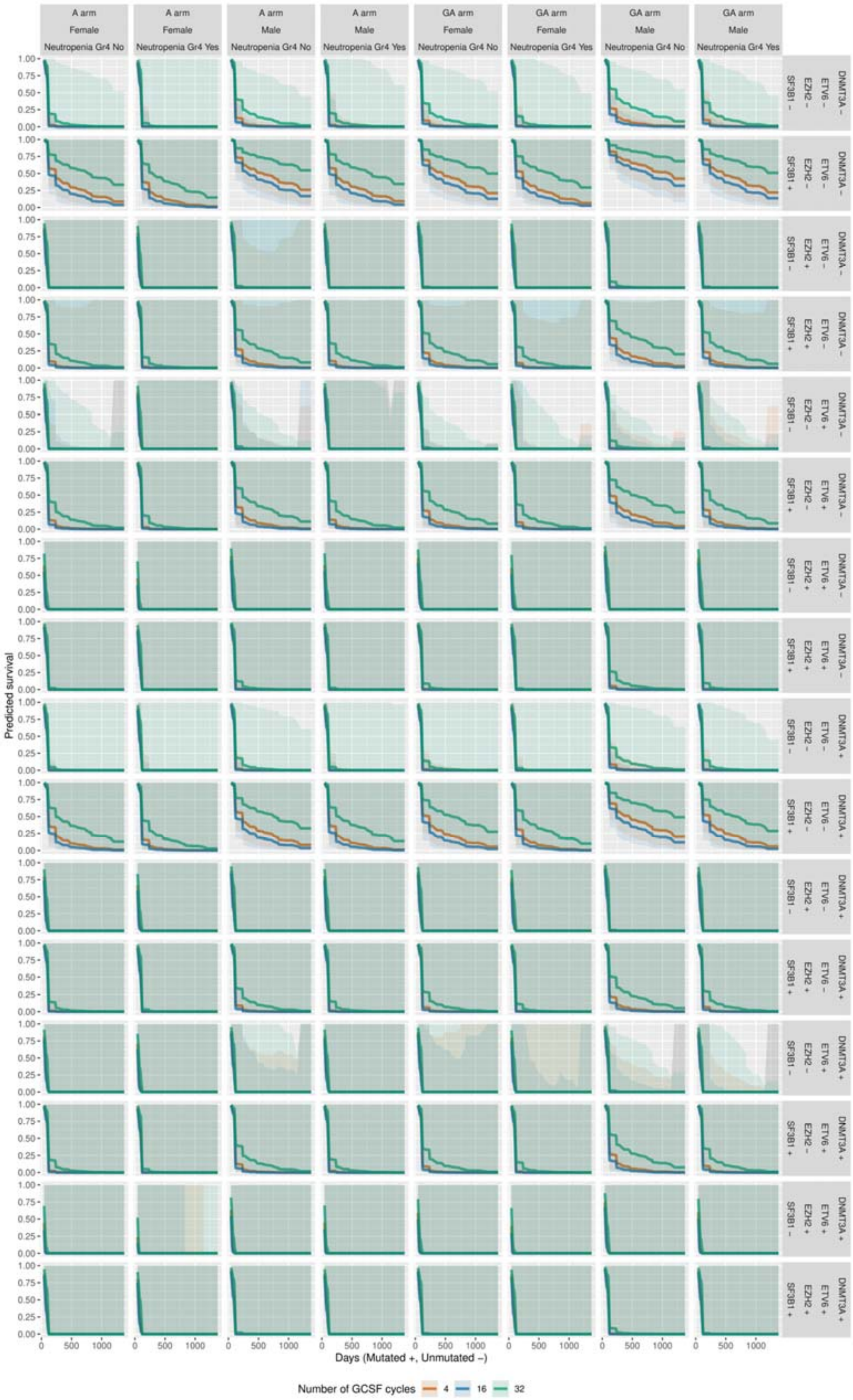

Supplementary Material SM17: Response to therapy in GA (blue) vs A (red) arm. Responses are: CR, PR, SD+HI. PD represents progression. Data are shown as a number of patients with particular response and in % for each restaging timepoint upon completed cycle 4, 8, & 12 with p-values from the multivariate joint stochastic model that should be considered as the results of the statistical analysis (due to multiple testing).

**Overall**	**GA (N=39)**	**A (N=31)**	**Total (N=70)**	**p value**
**ORR**	28 (72%)	14 (46%)	42 (60%)	0.000899
CR	12 (31%)	7 (23%)	19 (27%)	0.575
PR	9 (23%)	7 (23%)	16 (23%)	0.554
SD+HI	7 (18%)	0	7 (10%)	0.473
**Response C4**	**GA (N=39)**	**A (N=31)**	**Total (N=70)**	**p value**
CR	12 (31%)	6 (19%)	18 (26%)	0.4179
PR	8 (21%)	8 (26%)	16 (23%)	0.8123
SD+HI	8 (21%)	0	8 (11%)	0.0213
SD	3 (8%)	4 (13%)	7 (10%)	0.7483
PD	5 (13%)	7 (23%)	12 (17%)	0.4490
N/A	3 (8%)	6 (19%)	9 (13%)	0.2763
**Response C8**	**GA (N=34)**	**A (N=21)**	**Total (N=55)**	**p value**
CR	11 (32%)	6 (29%)	17 (31%)	0.5638
PR	3 (9%)	3 (14%)	6 (11%)	1.0000
SD+HI	3 (9%)	0	3 (5%)	0.3249
SD	3 (9%)	4 (19%)	7 (13%)	0.7483
PD	7 (21%)	4 (19%)	11 (20%)	0.8060
N/A	7 (21%)	4 (19%)	11 (20%)	0.8060
**Response C12**	**GA (N=16)**	**A (N=12)**	**Total (N=28)**	**p value**
CR	7 (44%)	4 (33%)	11 (40%)	0.8060
PR	2 (13%)	2 (17%)	4 (14%)	1.0000
SD+HI	2 (13%)	0	2 (7%)	0.5775
SD	1 (6%)	2 (17%)	3 (11%)	0.8386
PD	4 (25%)	4 (33%)	8 (29%)	1.0000
N/A	0	0	0	1.0000

Supplementary Material SM18: Predicted response rates (progressive disease (PD)/stable disease (SD)/partial remission (PR)/complete remission (CR)) to GA/A therapy from the ordinal multilevel logistic mixed model under the conditions shown at the top and right of each bar graph.

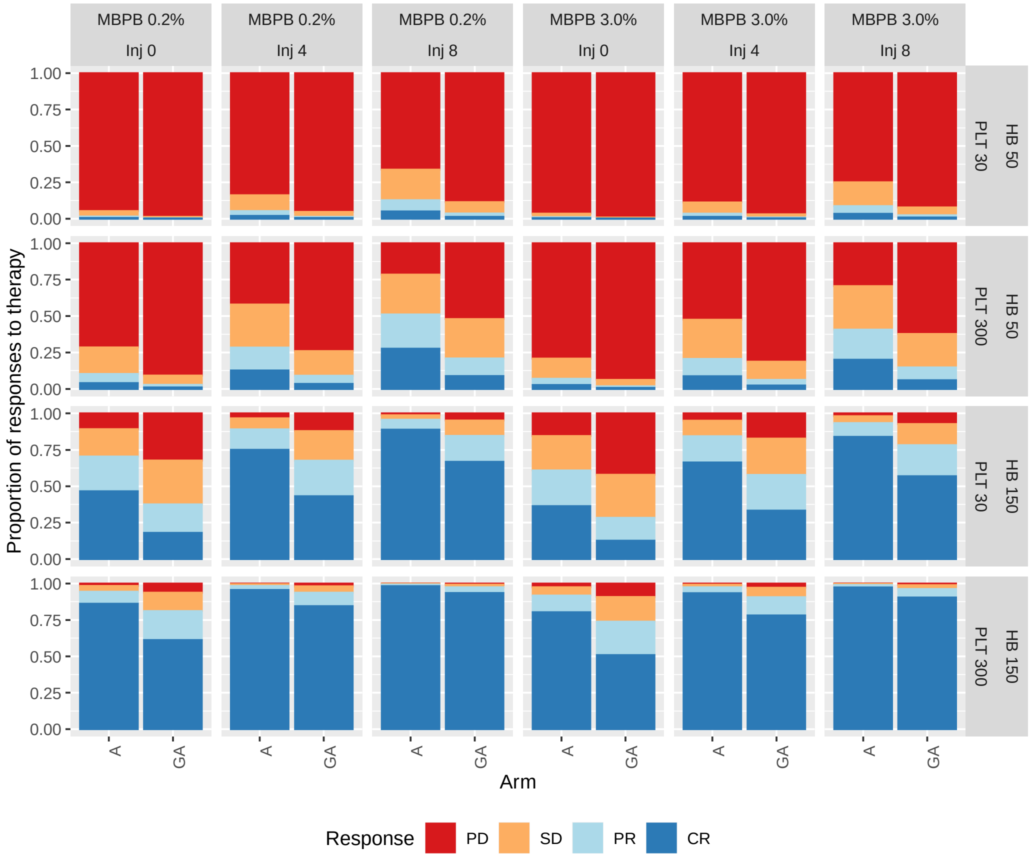

Supplementary Material SM19: Progression to AML was comparable in GA vs A arm. Numbers of patients are indicated (N).

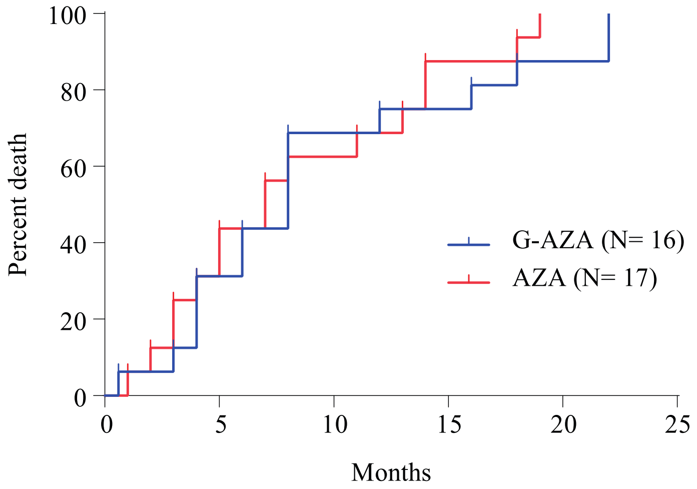

Supplementary Material SM20: A) Blood count parameters and biochemistry monitored each cycle in patients belonging to GA (blue) vs A (red) arm. T-test indicates negligible differences. B) Table indicates cytopenias, infections and infection-related mortality within the first 4 cycles of AZA.

A

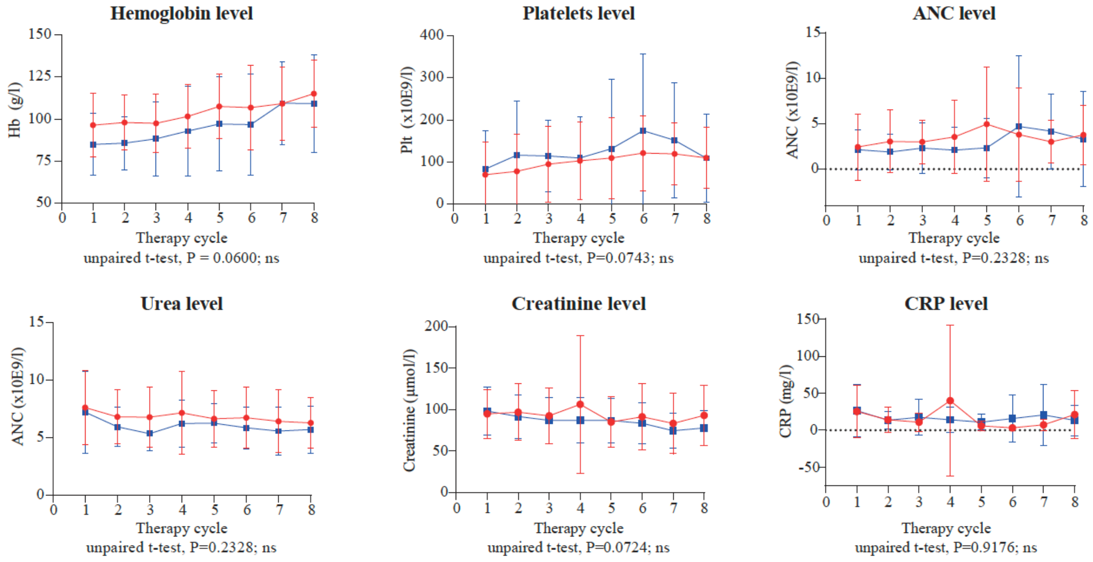

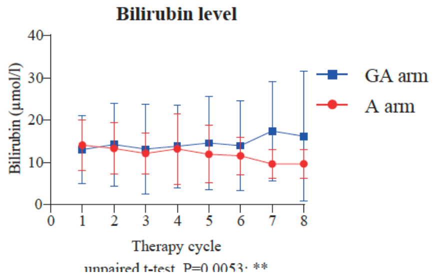

B

Initial 4 AZA cycles outcomes	Overal, N=70	A, N=31	GA, N=39
Reduction of AZA (N)	24 (34%)	9 (29%)	15 (38%)
TRF dependency (N)	41 (59%)	21 (68%)	20 (51%)
Gr>3 Anemia (N)	17 (24%)	14 (45%)	3 (8%)
Gr4 Thrombocytopenia (N)	11 (16%)	7 (23%)	4 (10%)
Gr4 Neutropenia observed (no infection) (N)	37 (53%)	12 (39%)	25 (64%)
Infection complications (N)	24 (34%)	11 (35%)	13 (33%)
FN, (38 °C, ANC<500/uL; N)	12 (17%)	2 (6%)	10 (26%)
Admission due to infection (Neutropenia<= Gr3; N)	9 (13%)	5 (16%)	4 (10%)
Admission due to FN (N)	5 (7%)	1 (3%)	4 (10%)
Infection-associated mortality (N)	12 (17%)	9 (29%)	3 (8%)

Supplementary Material SM21: Monitoring of cytopenias and infectious complications.
Regarding infections, during the initial 4 cycles of AZA, infections (such as most commonly pneumonia, uroinfections) were recorded in the GA vs A arm in 13 (33%) vs 11 (35%) patients. The number of patients hospitalized for infection was 4 (10%) in the GA arm vs 5 (16%) patients in the A arm, of which febrile neutropenia was noted in 10 (26%) vs 2 (6%) patients. 4 (10%, GA) vs 1 (3%, A) patients were admitted for FN. Infection-related mortality in the GA arm was 3 (representing 33% of GA patients who died within the first 4 cycles and 8% of all patients in the GA arm). Infection-related mortality in the A arm was 9 (representing 77% of all patients who died within the first 4 cycles and 29% of all patients in the A arm). Taken together, neutropenia was observed more frequently in the GA arm after the initial 4 cycles of AZA, which probably also reflects the number of neutropenic MDS patients entering (albeit incidentally) the GA arm, but this did not translate into any increase in infection-related mortality.

SUPPLEMENTAL REFERENCES:

1. Greenberg P, Cox C, LeBeau MM, Fenaux P, Morel P, Sanz G, et al. International scoring system for evaluating prognosis in myelodysplastic syndromes. Blood. 1997;89(6):2079-88.
2. Cheson BD, Greenberg PL, Bennett JM, Lowenberg B, Wijermans PW, Nimer SD, et al. Clinical application and proposal for modification of the International Working Group (IWG) response criteria in myelodysplasia. Blood. 2006;108(2):419-25.
3. Long JD, Mills JA. Joint modeling of multivariate longitudinal data and survival data in several observational studies of Huntington's disease. BMC Med Res Methodol. 2018;18(1):138.
4. Gijbels I, Omelka, M., Pešta, M., Veraverbeke, N. Score tests for covariate effects in conditional copulas. . Journal of Multivariate Analysis. 2017;159:111-33.
5. Rosner B. Fundamentals of Biostatistics (6-th edition), Thomson Brooks/Cole 2006(Section 14.12):807.
6. Freedman LS. Tables of the number of patients required in clinical trials using the logrank test. Stat Med. 1982;1(2):121-9.
7. Polgarova K, Vargova K, Kulvait V, Dusilkova N, Minarik L, Zemanova Z, et al. Somatic mutation dynamics in MDS patients treated with azacitidine indicate clonal selection in patients-responders. Oncotarget. 2017;8(67):111966-78.
8. Koboldt DC, Zhang Q, Larson DE, Shen D, McLellan MD, Lin L, et al. VarScan 2: somatic mutation and copy number alteration discovery in cancer by exome sequencing. Genome Res. 2012;22(3):568-76.
